# Temperature can reverse sexual conflict, facilitating population growth

**DOI:** 10.1093/evlett/qraf022

**Published:** 2025-08-01

**Authors:** Roberto García-Roa, Francisco Garcia-Gonzalez, Víctor Maroto, Valeria Chirinos, Ana Márquez-Rosado, Maider Iglesias-Carrasco, Pau Carazo

**Affiliations:** Department of Biology, Lund University, Lund, Sweden; Behaviour and Evolution Group, Ethology lab, Cavanilles Institute of Biodiversity and Evolutionary Biology, University of Valencia, Valencia, Spain; Evolution and Ecology of Sexual Interactions Group, Doñana Biological Station—CSIC, Seville, Spain; Centre for Evolutionary Biology, School of Biological Sciences, University of Western Australia, Crawley, Australia; Behaviour and Evolution Group, Ethology lab, Cavanilles Institute of Biodiversity and Evolutionary Biology, University of Valencia, Valencia, Spain; Behaviour and Evolution Group, Ethology lab, Cavanilles Institute of Biodiversity and Evolutionary Biology, University of Valencia, Valencia, Spain; Behaviour and Evolution Group, Ethology lab, Cavanilles Institute of Biodiversity and Evolutionary Biology, University of Valencia, Valencia, Spain; Department of Ecology, Evolution and Behaviour, University of Liverpool, Liverpool, United Kingdom; Evolution and Ecology of Sexual Interactions Group, Doñana Biological Station—CSIC, Seville, Spain; Globe Institute, University of Copenhagen, Copenhagen, Denmark; Behaviour and Evolution Group, Ethology lab, Cavanilles Institute of Biodiversity and Evolutionary Biology, University of Valencia, Valencia, Spain

**Keywords:** *Callosobruchus*, insects, male harm, sexual selection, sexual conflict, temperature

## Abstract

Sexual conflict frequently gives rise to adaptations that increase male reproductive success at the expense of harming females (“male harm”) and decreasing population growth. Studying the ecology of male harm is paramount to understand how sexual conflict unfolds in nature, and its consequences for populations viability. Here, we used seed beetles (*Callosobruchus maculatus*), a species where males harm females via both harassment and traumatic male insemination, to study whether temperature (24, 28, or 32 °C) can modulate male harm. We disentangled temperature effects on male harm via pre- and postcopulatory mechanisms (“harassment and mating”) vs. precopulatory mechanisms (i.e., “harassment” only; ablated males). These treatments were applied under different levels of sexual conflict, with females continuously exposed throughout their lifespan to either no males (control; “no harm”), 1 male (low sexual conflict), or 2 males (high sexual conflict). Constant exposure to males decreased female fitness at warmer environments, particularly at 28 °C and when females were subject to constant harassment and mating under high sexual conflict. In contrast, constant exposure to male harassment and mating increased female fitness at 24 °C, particularly under low sexual conflict (significant ~14% increase vs. control females). At the population level, not being exposed constantly to males resulted in higher net reproductive rates at 28 and 32 °C, whereas constant male–female cohabitation resulted in optimal net reproductive rates at 24 °C, rescuing estimated population growth rate and thus reversing the cost/benefit balance of exposure to males. Our findings show that, by dictating the outcome of female fitness under constant male exposure, temperature can modulate sexual conflict to the point of reversing it and facilitating population growth. Our results support the emerging notion that environmental variation can significantly decrease overall levels of sexual conflict in nature.

## Introduction

Sexual selection can often lead to divergent evolutionary interests in the reproductive strategies of females and males, also known as sexual conflict (Arnqvist & Rowe, 2005; Parker, 1979). Intralocus sexual conflict refers to the process where traits under sexually antagonistic selection share the same loci in both sexes, while interlocus sexual conflict occurs when sexually antagonistic selection acts on different traits in males and females (Bonduriansky & Chenoweth, 2009; Tregenza et al., 2006). Sexual conflict can play a key role in processes of reproductive isolation (Gavrilets, 2014), and may also result in detrimental effects for populations. For instance, intralocus sexual conflict pushes the sexes away from their evolutionary optima (Arnqvist & Tuda, 2009; Berger et al., 2016), while interlocus sexual conflict can hamper population growth via male harm to females; i.e., when male adaptations that allow them to outcompete their rivals over access to reproduction come at the cost of harming female fitness and, therefore, net population productivity (Gómez-Llano et al., 2024). Ultimately, this can lead to a “reproductive tragedy of the commons” (Rankin et al., 2007, 2011), increasing extinction risk over ecological or evolutionary time (Flintham et al., 2023; Le Galliard et al., 2005).

Male harm to females (hereafter “male harm”) is widespread across the tree of life (Gómez-Llano et al., 2024). Examples include male coercion (e.g., forced matings) in primate species, where females are often dominated due to their smaller size, the development of structures such as spiny penises, which damage females by causing injuries on their reproductive tract, or the indiscriminate harassment of numerous males upon a female appearing in the reproductive arena, as seen in several insects and amphibians (Clutton-Brock & Parker, 1995). As a countermeasure, females evolve adaptations to better resist male harm, giving rise to sexually antagonistic coevolution, an evolutionary arm race between males and females (Rice a& Holland, 1997) that can lead to significant genetic divergence among allopatric populations (Gavrilets, 2000, 2014; Parker & Partridge, 1998).

In recent decades, male harm has garnered significant attention. The vast diversity of male harm strategies observed in nature raises a pivotal question: How does the ecology of organisms shape the dynamics of male harm (Perry & Rowe, 2018; Plesnar‐Bielak & Łukasiewicz, 2021)? Among environmental factors, temperature emerges as particularly relevant due to its demonstrated potential to modulate the metabolism, physiology, and behavior of organisms (Bicego et al., 2007; Llusia et al., 2013; Nowakoski et al., 2018; Parret & Knell, 2018; Punzalan et al., 2008). In doing so, recent work has stressed that temperature can influence sexually selected traits both directly (e.g., by affecting their fitness outcomes, heritability, and developmental costs) and indirectly (e.g., by modulating the sex-specific costs and benefits of reproduction, reproductive rates, and/or female and male spatiotemporal distributions), making it a key abiotic factor to consider in sexual conflict studies (García-Roa et al., 2020). Still, our understanding of how the thermal environment during reproduction influences male harm is very limited (García-Roa et al., 2019; Londoño-Nieto et al., 2023, 2025). In the vinegar fly *Drosophila melanogaster*, recent evidence suggests that mechanisms of pre- and postcopulatory male harm are differently affected by temperature, and as a result net male harm is drastically modulated by the thermal environment (Londoño-Nieto et al., 2023, 2025). If widespread, these effects would be fundamental to understanding how sexual conflict unfolds in nature and what are its net consequences for populations. This study investigates whether changes in temperature influence male harm in another classical model species in the study of sexual conflict, the seed beetle *Callosobruchus maculatus* (e.g., Berger et al., 2016; Crudgington & Siva-Jothy, 2000; Iglesias-Carrasco et al., 2018a; McNamara et al., 2020; Zuk et al., 2014).

*Callosobruchus maculatus* is a cosmopolitan pest of leguminous plants. Adapted to predominantly warm environments typical of tropical and subtropical regions in Asia and Africa, its optimal temperature is on average around 28–30 °C (Beck & Blumer, 2014), with stock cultures at the laboratory often ranging from 25 to 35 °C (Beck & Blumer, 2014; Mahgoup at al. et al., 2019). At a 28–29 °C environment, adults emerge from beans after around 20 days of egg-to-adult development and live approximately 10–14 days. Adults do not require water or food for survival and reproduction, and males invest much effort in harassing females and mating. Males and females mate multiple times and both sexes exhibit secondary sexual traits. Notably, males have an aedeagus with sclerotized spines that puncture the reproductive tract of females during copulation (Crudgington & Siva-Jothy, 2000; Eady et al., 2007). Males with longer spines have higher relative reproductive success because these structures facilitate sperm penetration into the female reproductive tract (Hotzy & Arnqvist, 2009). Mating results in direct damage for females, in the form of decreased lifespan and reproductive output (den Hollander & Gwynne, 2009; Eady et al., 2007). Male harm intensifies in populations with a high relative density of males, as this increases male–male competition and elevates the pressure on females, for example through higher rates of male harassment (Berger et al., 2016). As a counteradaptation to male manipulative adaptations, females have evolved thick walls in their reproductive tracts (Dougherty et al., 2017; Rönn et al., 2007), as well as resistance behaviors, such as repeatedly shaking their hind legs to fend off males (Van Lieshout et al., 2014). Yet, mating also provides physiological benefits to *C. maculatus* females, as the water of male ejaculates can increase female fecundity (e.g., Edvarsson, 2007; Fox, 1993). Thus, males have evolved mating strategies that represent for females an interesting trade-off of reproductive costs and benefits (Eady et al., 2007; Edvardsson & Tregenza, 2005). Such cost–benefit balance is likely to be modulated by temperature changes because, first, temperature is bound to affect female hydric balance in the system, and second, as stated, recent evidence has shown that male harm mechanisms can be optimized at different temperatures (Londoño-Nieto et al., 2023, 2025).

**Figure 1.**
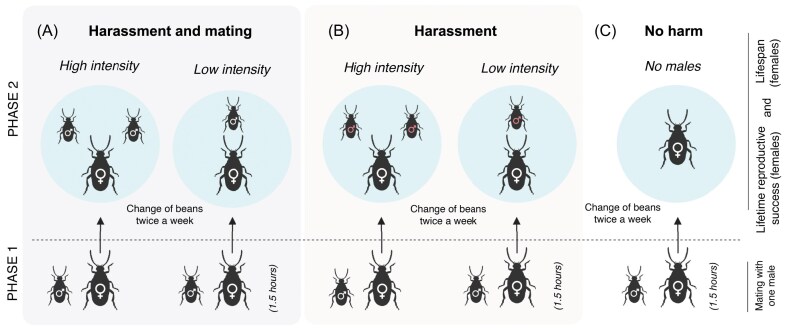
Experimental design of seed beetle (*Callosobruchus maculatus*) groups used in our study. In Phase 1, one experimental male was introduced alongside each virgin focal female for 1.5 hr at 28 °C, temperature at which the original population is maintained in the laboratory. In Phase 2, males were discarded and focal females were placed in Petri dishes to initiate different experimental treatments: (A) “harassment and mating” (low- or high-intensity; one female with one or two males, respectively); (B) “harassment” (low- or high-intensity; one female with one or two ablated males, respectively); and (C) “no harm” (females alone; i.e., control females). All these treatments were exposed at three different thermal scenarios: 24, 28, or 32 °C. Lifetime reproductive success and lifespan of females were measured. Mortality was checked twice a week (dead males were replaced by males of the same age), at which point all beans were changed by new ones. The inoculated beans by females were collected to start their incubation at 28 °C.

Here, we explored this idea by studying whether temperature changes, within the natural thermal range of *C. maculatus* (24, 28, or 32 °C), can modulate male harm through pre- and postcopulatory mechanisms (“harassment and mating”) or just precopulatory mechanisms (i.e., “harassment” only; ablated males) in females continuously exposed to either one (low sexual conflict) or two (high sexual conflict) males across their lifespan, vs. once-mated females alone (“no harm”; i.e., control) (Figure 1).

## Materials and methods

### Stock culture and maintenance

The seed beetles originated from a large laboratory outbred population (south Indian stock population), harboring high phenotypic and genetic variability (e.g., see Canal et al., 2022; Rodriguez-Exposito & Garcia-González, 2021). With beetles from this population, we set up a new stock population at the University of Valencia (Valencia, Spain), which was maintained at a population size in excess of 300 individuals, under nonoverlapping generations, and in a constant temperature incubator of 28 °C temperature, 40% humidity, and a 12 hr/12 hr light/dark cycle, with ad libitum organic mung beans (*Vigna radiata*) serving as their culture medium.

### Experimental design

In order to study whether temperature variation modulates male harm through pre- and postcopulatory mechanisms (“harassment and mating” treatment) or just precopulatory mechanisms (“harassment” treatment), we subjected experimental females to either one (low sexual conflict) or two (high sexual conflict) males across females lifespan, vs. females that were kept alone (“no harm” treatment; control), at a thermal environment of 24, 28, or 32 °C. We measured the outcome in terms of female lifetime reproductive success.

To set up these assays, 50 females (F0) from the stock population (i.e., mated, as they were with males) were haphazardly selected and transferred into individual Petri dishes containing 100 beans for egg laying. The isolation of each female in one Petri dish allowed us to better control the laying environment, ensuring that all eggs had the same developmental time and controlled for density effects. After a 24-hr period, beans with only one egg were individually isolated in Eppendorf (as in previous studies; see, e.g., Castano-Sanz et al., 2024; Rodriguez-Exposito & Garcia-Gonzalez, 2021) tubes and incubated at 28 °C. From these beans, we collected virgin females and males for the experiment (F1: 750 focal females and 1,500 experimental males, along with 300 extra males to replace potential dead males across the experiment), which we haphazardly allocated to different treatments (see later).

The experiment started with all focal virgin females being isolated in Eppendorf tubes with a single virgin male at 28 °C for 1.5 hr (Figure 1) to ensure all females across treatments had mated at least once (e.g., Castano-Sanz et al., 2024; Eady, 1995; Savalli & Fox, 1999). In parallel, we cut the aedeagus to half of experimental virgin males using surgical scissors after anesthesia with CO_2_ (Jigisha et al., 2020; Zajitschek et al., 2018). This results in ablated males that can court and harass females, but not mate with them. We anesthetized the other half of experimental virgin unablated males to ensure they experienced similar conditions (apart from ablation). We used ablated males (without aedeagus) for the “harassment” treatment and nonablated males (with aedeagus) for the “harassment and mating” treatment (see Figure 1).

We started fitness assays by combining focal females with experimental males that we haphazardly allocated to the following sexual conflict treatments factorially combined across three temperatures (24, 28, or 32 °C): (a) “harassment and mating” (low- or high-intensity sexual conflict; one female with one or two males, respectively); (b) “harassment” (low- or high-intensity sexual conflict; one female with one or two ablated males, respectively); and (c) “no harm” (females alone; i.e., control females) (Figure 1). Sample sizes across treatments averaged 41 (37–49) replicates (see Supplementary Material for sample size). Each experimental group of beetles was introduced into a Petri dish (i.e., the reproductive arena of each focal female) with 100 beans, which we replaced by new ones twice a week. Dead males were replaced by new ones of the same age. Females were checked twice a week for survival until their death, and the inoculated beans were incubated at 28 °C until adult emergence, to estimate female lifespan and female lifetime reproductive success, respectively. Note that there may be slight differences in developmental time caused by temperature differences during the first 3–4 days (i.e., the time beans remained at their respective temperature treatments before putting them at the common incubation temperature). Nonetheless, because we waited until full adult emergence and, more importantly, given that these potential differences in development were consistent across temperatures within each sexual conflict treatment, they are highly unlikely to introduce any biases in our results.

As an indirect measure of how male harm may impact population growth/viability, we calculated the net reproductive rate of female cohorts under “no harm” (control) and “harassment and mating” and “harassment” (both under low- and high-intensity sexual conflict) treatments as *R*_0_ = Σ lx × mx: the sum of the lx (the proportion of surviving females at the beginning of age class X) and mx (the number of living individuals born per female in each interval class).

### Statistical analyses

We fitted linear models to examine the effects of temperature, sexual conflict treatment, and their interaction, as fixed factors, on female lifetime reproductive success and lifespan using lme4 (Bates et al., 2015). To gain power and focus on overall differences across treatments due to the type of sexual conflict females were exposed to (i.e., whether females were subject to harassment and mating or only harassment), we conducted a second set of analyses after pooling data from low and high intensity of sexual conflict within the “harassment and mating” and “harassment” treatments. Therefore, this second set of analyses only had three sexual conflict treatments: (a) harassment and mating (females mated once as virgins and then exposed to continuous harassment and mating by one or two males), (b) harassment only (females mated once as virgins to then being continuously harassed by one or two ablated males), and (c) no harm (females alone, mated once as virgins and isolated thereafter). Due to the presence of potential outliers in our data (Figure S1), lifetime reproductive success and lifespan variables were transformed through alpha-winsorization, a robust statistical method where extreme values at alpha > 0.05 are replaced by the next most extreme value within the remaining distribution of data, thus ensuring we control for the undue influence of these values while retaining their rank position within the distribution; i.e., no data points were removed. We then proceeded to transform values to *z*-scores, so as to reliably interpret principal effects in models with significant interactions (Schielzeth, 2010). Graphs were created with ggplot2 (Wickham et al., 2016). All analyses were conducted in R.3.2.4 (R Core Team., 2014).

## Results

**Figure 2.**
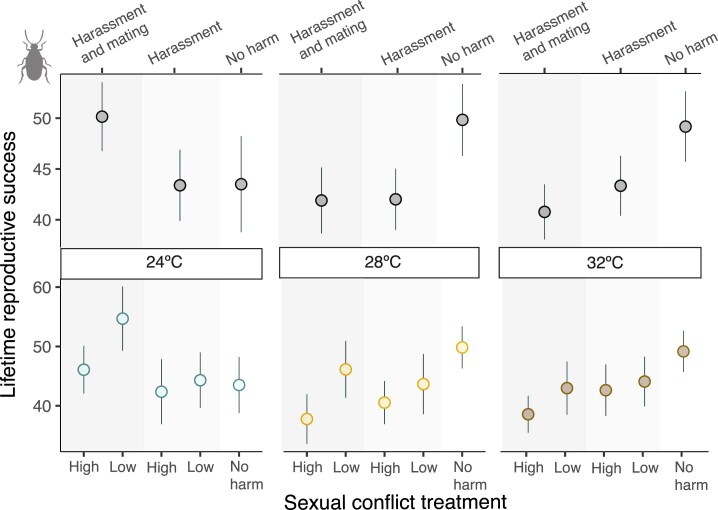
Plots for lifetime reproductive success of *Callosobruchus maculatus* females (mean ± SEM) exposed at three different temperatures (24, 28, or 32 °C). The lifetime reproductive success according to the sexual conflict treatment (“harassment and mating,” “harassment,” and “no harm”; top) and its intensity (high-intensity: two males and one female; low-intensity: one male and one female; and no harm: females alone; bottom). Top plot results from the combination of intensity treatments. All focal females had a preliminary stage in which they were allowed to mate with one male.

We found a significant interaction between temperature and sexual conflict treatment (i.e., high-intensity “harassment and mating,” low-intensity “harassment and mating,” high-intensity “harassment,” and low-intensity “harassment” and “no harm”) on female lifetime reproductive success (*F*_8,576_ = 3.292, *p =* 0.001), with a significant main effect of sexual conflict treatment (*F*_4,576_ = 6.692, *p <* 0.001), but not of temperature (*F*_2,576_ = 1.603, *p =* 0.202) (Figure 2 bottom plot). To explore this interaction, we then ran models separately for each temperature and found that sexual conflict treatment affected female lifetime reproductive success at the three temperatures. At 28 °C (*F*_4,196_ = 5.588, *p <* 0.001, estimate: −0.4895 ± 0.1512), this effect was driven by female lifetime reproductive success being lower under high-intensity “harassment and mating” compared to the low-intensity “harassment and mating” and “no harm” treatments (Figure 2 bottom plot; Table 1). At 32 °C (*F*_4,191_ = 4.233, *p =* 0.002, estimate: −0.4307 ± 0.1384), females had a lower lifetime reproductive success when exposed to high-intensity “harassment and mating” vs. “no harm” treatment (Figure 1; Table 1). In contrast, at 24 °C (*F*_4,189_ = 3.598, *p =* 0.007, estimate: 0.1222 ± 0.1696), female lifetime reproductive success was not significantly different, under high-intensity “harassment” or “harassment and mating” than under “no harm,” and actually increased significantly under low-intensity “harassment and mating” compared to both low-intensity and high-intensity “harassment” and “no harm” (Figure 2 and Table 1). When we explored the potential interaction between temperature and sexual conflict treatment with lifespan as a covariable, we obtained similar effects; the interaction remained significant (*F*_8,575_ = 2.992, *p =* 0.002), with main effects arising only from the sexual conflict treatment (*F*_4,575_ = 6.741, *p <* 0.001), and not from temperature (*F*_2,575_ = 1.615, *p =* 0.199).

**Table 1.** Results from post hoc tests assessing potential differences in the lifetime reproductive success of *Callosobruchus maculatus* females across temperatures and sexual conflict treatments.

Lifetime reproductive success	24 °C			28 °C			32 °C		
	Estimate	*t*	*p*	Estimate	*t*	*p*	Estimate	*t*	*p*
Harassment/harassment and mating	**−0.460 ± 0.161**	**−2.863**	**0.012**	0.006 ± 0.158	0.037	0.999	0.183 ± 0.160	1.144	0.488
Harassment/no harm	0.016 ± 0.183	0.089	0.996	**−0.563 ± 0.181**	**−3.112**	**0.006**	*−0.411 ± 0.185*	*−2.228*	*0.067*
Harassment and mating/no harm	**0.476 ± 0.183**	**2.596**	**0.026**	**−0.569 ± 0.179**	**−3.184**	**0.004**	**−0.595 ± 0.179**	**−3.315**	**0.003**
Harassment and mating_High_/harassment and mating_Low_	−0.565 ± 0.225	−2.514	0.089	**−0.625 ± 0.217**	**−2.875**	**0.034**	−0.334 ± 0.216	−1.547	0.532
Harassment and mating_High_/harassment_High_	0.259 ± 0.225	1.153	0.778	−0.203 ± 0.217	−0.934	0.884	−0.298 ± 0.224	−1.332	0.672
Harassment and mating_High_/harassment_Low_	0.132 ± 0.219	0.603	0.975	−0.438 ± 0.224	−1.961	0.287	−0.405 ± 0.225	−1.797	0.376
Harassment and mating_High_/no harm	0.208 ± 0.210	0.991	0.859	**−0.877 ± 0.207**	**−4.243**	**0.000**	**−0.762 ± 0.208**	**−3.667**	**0.003**
Harassment and mating_Low_/harassment_High_	**0.825 ± 0.231**	**3.572**	**0.004**	0.422 ± 0.219	1.929	0.303	0.037 ± 0.224	0.163	1.000
Harassment and mating_Low_/harassment_Low_	**0.697 ± 0.225**	**3.100**	**0.017**	0.187 ± 0.225	0.829	0.922	−0.071 ± 0.225	−0.314	0.998
Harassment and mating_Low_/no harm	**0.774 ± 0.217**	**3.571**	**0.004**	−0.253 ± 0.208	−1.213	0.744	−0.428 ± 0.208	−2.059	0.240
Harassment_High_/harassment_Low_	−0.128 ± 0.225	−0.567	0.980	−0.235 ± 0.225	−1.046	0.834	−0.107 ± 0.233	−0.461	0.991
Harassment_High_/no harm	−0.051 ± 0.217	−0.235	0.999	**−0.675 ± 0.208**	**−3.239**	**0.011**	−0.464 ± 0.216	−2.153	0.200
Harassment_Low_/no harm	0.077 ± 0.210	0.364	0.996	−0.439 ± 0.215	−2.045	0.246	−0.357 ± 0.217	−1.642	0.471

*Note*. Significant results are highlighted in bold, and marginally nonsignificant results are highlighted in italics. High: two males and one female (high intensity of sexualconflict; Low: one male and one female (low intensity of sexual conflict).

We then analyzed our data after pooling low- and high-intensity sexual conflict treatments within the “harassment and mating” and “harassment” treatments (i.e., resulting in only three sexual conflict treatments: “harassment and mating,” “harassment,” and “no harm”), and obtained qualitatively similar results (Figure 2, top plot). We detected a significant interaction between temperature and sexual conflict treatment (*F*_4,582_ = 5.992, *p <* 0.001; Figure 2), as well as a significant main effect of sexual conflict treatment (*F*_2,582_ = 4.899, *p =* 0.007), but not of temperature (*F*_2,582_ = 1.569, *p =* 0.209). To explore this interaction, we ran separate analyses for each temperature and found that sexual conflict treatment influenced the lifetime reproductive success of females across all temperatures. At 28 °C (*F*_2,198_ = 6.168, *p =* 0.002, estimate: −0.175 ± 0.113), females had a lower lifetime reproductive success under “harassment and mating” and “harassment” than under “no harm” (with not differences between “harassment” and “harassment and mating”; Figure 2 top plot; Table 1). At 32 °C (*F*_2,193_ = 6.837, *p =* 0.001, estimate: −0.080 ± 0.105), females also had a lower lifetime reproductive success under “harassment and mating” than under “no harm,” and under “harassment” and “no harm” (but the later result was marginally nonsignificant; Table 1). In contrast, females at 24 °C (*F*_2,191_ = 4.352, *p =* 0.014, estimate: −0.069 ± 0.124) had a higher lifetime reproductive success under “harassment and mating” than under both “harassment” or “no harm” (Figure 2 and Table 1). We also explored whether the interaction remained significant when including lifespan as a covariable. Indeed, we found a significant temperature by treatment interaction (*F*_4,581_ = 5.433, *p <* 0.001), along with a significant main effect of treatment (*F*_2,581_ = 4.964, *p =* 0.007), but not of temperature (*F*_2,581_ = 1.59, *p =* 0.204). See Supplementary Material for results on females’ lifespan (Table S1 and Figure S2).

## Discussion

**Figure 3.**
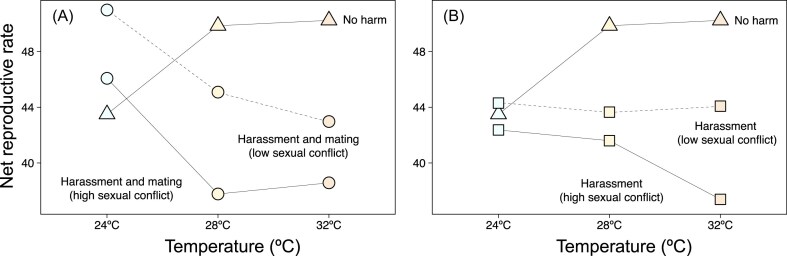
Net reproductive rate of seed beetle *Callosobruchus maculatus* females that are subject to “no harm” (triangles) or are subject to “harassment and mating” (circles) or “harassment” (squares) of one (low sexual conflict; dashed line); or two males (high sexual conflict), across the three experimental thermal environments: 24, 28, and 32 °C. Panel (A) shows the comparison between the “no harm” and “harassment and mating” treatments, and panel (B) shows the comparison between the “no harm” and “harassment” treatments. Net reproductive rate was calculated as the sum of the lx (the proportion of surviving individuals at the beginning of age class) and mx (the number of living individuals born per female in each interval class), following equation: *R*_0_ = Σ lx × mx. Values hence represent aggregated population-level estimates, with no applicable error bars.

We show that temperature acts as a modulator of sexual conflict via male harm in *C. maculatus*. At 28 °C, increased exposure to males generally imposed fitness costs for females regardless of the mechanism of harm involved (i.e., harassment and mating or only harassment). This trend persisted, albeit less markedly, in the warmest environment (32 °C). In stark contrast, at 24 °C neither constant harassment nor constant harassment and mating decreased female fitness. On the contrary, continuous harassment and mating significantly increased female fitness in low-intensity sexual conflict, resulting in a higher net reproductive rate (Figure 3). Overall, we show that temperature variation determines sexual conflict by modulating the cost/benefit balance of male exposure for females and, in doing so, may facilitate population growth at low temperatures.

The highest costs of male harm (i.e., the largest decrease in female lifetime reproductive success) occurred at 28 °C, followed by 32 °C (Figure 2 and 3). *Callosobruchus maculatus* thrives at moderately elevated temperatures (28–34 °C) (Beck & Blumer, 2014), with thermal stress reported above 35–37 °C (e.g., Daglish et al., 2021; Iglesias et al., 2024; Lale & Vidal, 2003; Mahgoup et al., 2019). It is, therefore, perhaps not surprising that male harm was higher in the two warmer environments, peaking at the temperature at which our studied population is maintained in the laboratory (28 °C). In contrast, the costs of male harm seemed to vanish at lower temperatures (24 °C), with a clear increase in female lifetime reproductive success when females were exposed to a “harassment and mating” treatment under low-intensity sexual conflict. This resulted in interesting population dynamics. In the absence of harm, estimated population growth (i.e., net reproductive rate) was clearly higher at 28 and 32 °C than at 24 °C, again in accordance with the idea that *C. maculatus* females are better adapted to reproduce at high temperatures (Beck & Blumer, 2014; Chandrantha et al., 1987; Daglish et al., 2021). Interestingly, though, constant exposure to males (i.e., constant harassment and mating, which is the natural sociosexual context for this species in the wild) resulted in an increase in the net reproductive rate at 24 °C that was equal (or even slightly higher) to the maximum reproductive rates under no harm at both 28 and 32 °C. This suggests that the cost/benefit balance of exposure to males is reversed at low temperatures, resulting in optimal population growth rates despite being exposed to a potentially maladaptive temperature. Several complementary phenomena may contribute to explain these results.

First, males may be less harmful when exposed to low temperatures, for example, via a reduction in the harassment of females due to temperature effects on male activity rates and/or metabolism. Consistent with this hypothesis, in *D. melanogaster* male harassment rates and ensuing male harm drastically decrease at lower temperatures, even when temperature changes are well within the optimal reproductive thermal range of the population (Londoño-Nieto et al., 2023, 2025). In addition to such thermally plastic responses, male flies from this same species evolve to exhibit lower levels of harassment, and to be less thermally plastic, when locally adapting to cold temperatures (Londoño-Nieto et al., 2025). The parallelism in both plastic and evolutionary responses suggests that low temperatures impose low levels of male harassment to females (e.g., via shared constraints and/or changes in the costs associated with male activity). This also fits well with our results in that we did not find any effects of harassment per se on female fitness at 24 °C (Figure 2 and Table 1). Nonetheless, further research is needed to ascertain whether male harming behavior in *C. maculatus* may also vary in response to the thermal environment, and to elucidate the mechanisms at play. Importantly, it is worth noting that, even at 24 °C, under constant male exposure to females (harassment and mating) we found a clear (albeit not significant) trend for female fitness to be lower in high- vs. low-intensity sexual conflict. This is in accordance with prior studies and suggests that increased sexual conflict does in fact lead to higher male harm even at cold temperatures (e.g., den Hollander & Gwynne, 2009; Edvarsson et al., 2007).

Second, population growth and viability depend not only on male ability to inflict harm, but also on female resistance to harm (Gay et al., 2011; Rankin et al., 2007, 2011). Harm resistance in *C. maculatus* females consists of a suite of behavioral, physiological, and morphological strategies, such as kicking the males during mating, developing efficient immune responses to prevent infection from copulatory wounding, and reinforcing reproductive tracts to minimize injuries by the spiny aedeagus (Dougherty et al., 2017; Eady et al., 2007; Röon et al., 2007). Female adaptations for resistance have been shown to co-evolve quickly in response to ecology (Perry et al., 2017; Rodriguez-Exposito & Garcia-Gonzalez, 2021). Female adaptive plasticity in resistance is expected to evolve (McLeod & Day, 2017), and plastic effects influenced by temperature are, in principle, equally as likely to happen for female resistance than for male harm. Thus, it is easy to conceive that temperature changes may trigger both adaptive and maladaptive responses in female resistance, which will affect the net outcome of sexual conflict via male harm. However, our experiment cannot determine whether females are better at resisting male harm at lower temperatures, nor identify which, if any, underlying mechanisms may be involved. Epigenetic mechanisms are probably also involved, as elucidated by recent research showing that epigenetic responses are implicated in modulating temperature-related life-history trajectories in *C. maculatus* (McCaw et al., 2024). We suggest that future research should flesh out thermal effects on female resistance vs. male harm ability in this and other systems.

Third, the costs of male harassment and mating harm may be offset, or even reversed, by benefits associated with mating (Reinhardt et al., 2009). In *C. maculatus*, ejaculates are a source of water and nutrition for females. Females receiving larger ejaculates have higher lifetime reproductive success (Edvardsson & Canal, 2006; Savalli & Fox, 1999), and these effects are largely mediated by the water content in the ejaculate, which helps females avoid dehydration and withstand the costs of reproduction (Edvardsson, 2007; Ursprung et al., 2009). Increasing the amount of water in the ejaculate can also benefit males, as reducing the female need for water can reduce female remating (Edvardsson, 2007), and hence the risk of sperm competition, by allowing females to wait longer until the next mating (Fox, 1993; Paukku & Kotiaho, 2005). However, this is not a cost-free strategy for males, especially in warmer environments, where metabolic demands and water evapotranspiration are higher (Harrison et al., 2012). Thus, lower constraints in colder environments might allow males to invest in larger and/or more aqueous ejaculates. In fact, males have been shown to prefer relatively cooler reproductive environments than females (Małek & Czarnoleski, 2021). Although speculative, a more copious ejaculate in cooler environments would provide more benefits to females that may help them counterbalance the costs of male harm. Interestingly, this also raises further questions about whether more prolonged thermal effects—such as those occurring during development—on phenotypic condition (Adhikary & Barik, 2012; Lale & Vidal, 2003) and reproductive traits (Vasudeva, 2014) in *C. maculatus* may play a role in shaping sexual conflict.

To conclude, there is a growing appreciation for the role that ecological factors play in modulating sexual selection and sexual conflict (Arbuthnott et al., 2014; De Lisle et al., 2018; Gomez‐Llano et al., 2018; Martinossi-Allibert et al., 2017; Perry & Rowe, 2018; Perry et al., 2017; Rodriguez-Exposito & Garcia-Gonzalez, 2021; Yun et al., 2017, 2018), and recent studies have shown that temperature might be a particularly important abiotic factor in this dynamic interplay (Berger et al., 2014; García-Roa et al., 2020; Londoño-Nieto et al., 2023, 2025). According to the last Intergovernmental Panel on Climate Change (IPCC, 2021), while global warming is indeed expected to raise average global temperatures, this does not rule out the occurrence of regional or temporal temperature drops, which may arise from the complex interplay of atmospheric and oceanic circulation patterns, jet stream dynamics, or land use changes. In this context, local decreases in temperature remain ecologically relevant scenarios, particularly in temperate regions where seasonal variability is pronounced. We here show that, in *C. maculatus*, relatively minor temperature changes cannot only buffer sexual conflict via male harm to females, but even reverse its net fitness effects on females, and potentially facilitate population growth. In addition, these results add to recent evidence to suggest that environmental variation and complexity may decrease overall levels of sexual conflict in nature (Berger et al., 2014; Londoño-Nieto et al., 2023, 2025; Yun et al., 2017, 2018). Our results highlight the importance of considering thermal ecology to understand how sexual conflict unfolds in the wild, and its consequences for female fitness and population viability, which may be particularly important in the face of rapid environmental changes.

## Supplementary Material

qraf022_Supplemental_File

## Data Availability

The data supporting this study are available in Dryad (10.5061/dryad.fn2z34v4h)

## References

[bib1] Adhikary P., Barik A. (2012). Effect of temperature on biology of *Callosobruchus maculatus* (F.). Indian Journal of Entomology, 74(3), 261–266.

[bib2] Arbuthnott D., Dutton E. M., Agrawal A. F., Rundle H. D. (2014). The ecology of sexual conflict: Ecologically dependent parallel evolution of male harm and female resistance in *Drosophila melanogaster*. Ecology Letters, 17, 221–228. 10.1111/ele.12222 24215269

[bib3] Arnqvist G., Rowe L. (2005). Sexual conflict. Princeton University Press. 10.1515/9781400850600

[bib4] Arnqvist G., Tuda M. (2009). Sexual conflict and the gender load: Correlated evolution between population fitness and sexual dimorphism in seed beetles. Proceedings of the Royal Society of London B, 277(1686), 1345–1352. 10.1098/rspb.2009.2026 PMC2871940 20031994

[bib5] Bates D., Mächler M., Bolker B., Walker S. (2015). Fitting linear mixed-effects models using lme4. Journal of Statistical Software, 67, 1–48. 10.18637/jss.v067.i01

[bib6] Beck C. W., Blumer L. S. (2014). A handbook on bean beetles, Callosobruchus maculatus(pp. 1–17). National Science Foundation.

[bib7] Berger D., Grieshop K., Lind M. I., Goenaga J., Maklakov A. A., Arnqvist G. (2014). Intralocus sexual conflict and environmental stress. Evolution, 68, 2184–2196. 24766035 10.1111/evo.12439

[bib8] Berger D., Martinossi-Allibert I., Grieshop K., Lind M. I., Maklakov A. A., Arnqvist G. (2016). Intralocus sexual conflict and the tragedy of the commons in seed beetles. The American Naturalist, 188, E98–E112. 10.1086/687963 27622882

[bib9] Bicego K. C., Barros R. C., Branco L. G. (2007). Physiology of temperature regulation: Comparative aspects. Comparative Biochemistry and Physiology Part A: Molecular & Integrative Physiology, 147(3), 616–639. 10.1016/j.cbpa.2006.06.032 16950637

[bib10] Bonduriansky R., Chenoweth S. F. (2009). Intralocus sexual conflict. Trends in Ecology & Evolution, 24, 280–288. 10.1016/j.tree.2008.12.005 19307043

[bib11] Canal D., Garamszegi L. Z., Rodriguez-Exposito E., Garcia-Gonzalez F. (2022). Experimental evolution reveals differential evolutionary trajectories in male and female activity levels in response to sexual selection and metapopulation structure. Evolution, 76(6), 1347–1359. 10.1111/evo.14499 35483712 PMC9320835

[bib12] Castano-Sanz V., Gomez-Mestre I., Rodriguez-Exposito E., Garcia-Gonzalez F. (2024). Pesticide exposure triggers sex-specific inter-and transgenerational effects conditioned by past sexual selection. Proceedings of the Royal Society B: Biological Sciences, 291(2027), 20241037. 10.1098/rspb.2024.1037 PMC11252676 39014998

[bib13] Chandrantha J., Muthukrishnan J., Mathavan S. (1987). Effect of temperature and host seed species on the fecundity of *Callosobruchus maculatus* (F.). Proceedings: Animal Sciences, 96, 221–227.

[bib14] Clutton-Brock T. H., Parker G. A. (1995). Sexual coercion in animal societies. Animal Behaviour, 49, 1345–1365. 10.1006/anbe.1995.0166

[bib15] Crudgington H. S., Siva-Jothy M. T. (2000). Genital damage, kicking and early death. Nature, 407, 855. 10.1038/35038154 11057654

[bib16] Daglish G. J., Jagadeesan R., Nayak M. K. (2021). Temperature-dependent development and reproduction of the cowpea weevil, *Callosobruchus maculatus* F., in mungbean: Estimating a target temperature for its control using aeration cooling. Journal of Stored Products Research, 92, 101815. 10.1016/j.jspr.2021.101815

[bib17] De Lisle S. P., Goedert D., Reedy A. M., Svensson E. I. (2018). Climatic factors and species range position predict sexually antagonistic selection across taxa. Philosophical Transactions of the Royal Society B: Biological Sciences, 373(1757), 20170415. 10.1098/rstb.2017.0415 PMC6125731 30150216

[bib18] den Hollander M., Gwynne D. T. (2009). Female fitness consequences of male harassment and copulation in seed beetles, *Callosobruchus maculatus*. Animal Behaviour, 78(5), 1061–1070.

[bib19] Dougherty L. R., van Lieshout E., McNamara K. B., Moschilla J. A., Arnqvist G., Simmons L. W. (2017). Sexual conflict and correlated evolution between male persistence and female resistance traits in the seed beetle *Callosobruchus maculatus*. Proceedings of the Royal Society B: Biological Sciences, 284(1855), 20170132. 10.1098/rspb.2017.0132 PMC5454259 28539510

[bib20] Eady P. E. (1995). Why do male *Callosobruchus maculatus* beetles inseminate so many sperm?. Behavioral Ecology and Sociobiology, 36, 25–32. 10.1007/BF00175725

[bib21] Eady P. E., Hamilton L., Lyons R. E. (2007). Copulation, genital damage and early death in *Callosobruchus maculatus*. Proceedings of the Royal Society B: Biological Sciences, 274(1607), 247–252. 10.1098/rspb.2006.3710 PMC1685841 17035168

[bib22] Edvardsson M. (2007). Female *Callosobruchus maculatus* mate when they are thirsty: Resource-rich ejaculates as mating effort in a beetle. Animal Behaviour, 74(2), 183–188. 10.1016/j.anbehav.2006.07.018

[bib23] Edvardsson M., Canal D. (2006). The effects of copulation duration in the bruchid beetle *Callosobruchus maculatus*. Behavioral Ecology, 17(3), 430–434. 10.1093/beheco/arj045

[bib24] Edvardsson M., Tregenza T. (2005). Why do male *Callosobruchus maculatus* harm their mates?. Behavioral Ecology, 16(4), 788–793. 10.1093/beheco/ari055

[bib25] Flintham E. O., Savolainen V., Mullon C. (2023). Male harm offsets the demographic benefits of good genes. Proceedings of the National Academy of Sciences, 120(10), e2211668120. 10.1073/pnas.2211668120 PMC10013744 36862690

[bib26] Fox C. W. (1993). Multiple mating, lifetime fecundity and female mortality of the bruchid beetle, *Callosobruchus maculatus* (Coleoptera: Bruchidae). Functional Ecology, 7, 203–208. 10.2307/2389888

[bib27] García-Roa R., Chirinos V., Carazo P. (2019). The ecology of sexual conflict: Temperature variation in the social environment can drastically modulate male harm to females. Functional Ecology, 33(4), 681–692. 10.1111/1365-2435.13275

[bib28] García-Roa R., Garcia-Gonzalez F., Noble D. W., Carazo P. (2020). Temperature as a modulator of sexual selection. Biological Reviews, 95(6), 1607–1629. 10.1111/brv.12632 32691483

[bib29] Gavrilets S. (2000). Rapid evolution of reproductive barriers driven by sexual conflict. Nature, 403, 886. 10.1038/35002564 10706284

[bib30] Gavrilets S. (2014). Is sexual conflict an “engine of speciation”?. Cold Spring Harbor Perspectives in Biology, 6, a017723. 10.1101/cshperspect.a017723 25395295 PMC4292158

[bib31] Gay L., Brown E., Tregenza T., Pincheira-donoso D., Eady P. E., Vasudev R., Hosken D. J. (2011). The genetic architecture of sexual conflict: Male harm and female resistance in *Callosobruchus maculatus*. Journal of Evolutionary Biology, 24(2), 449–456. 10.1111/j.1420-9101.2010.02182.x 21126275

[bib33] Gomez-Llano M. A., Bensch H. M., Svensson E. I. (2018). Sexual conflict and ecology: Species composition and male density interact to reduce male mating harassment and increase female survival. Evolution, 72, 906–915. 10.1111/evo.13457 29465798

[bib32] Gómez-Llano M., Faria G. S., García-Roa R., Noble D. W., Carazo P. (2024). Male harm suppresses female fitness, affecting the dynamics of adaptation and evolutionary rescue. Evolution Letters, 8(1), 149–160. 10.1093/evlett/qrac002 38370549 PMC10871930

[bib34] Harrison J. F., Woods H. A., Roberts S. P. (2012). Ecological and environmental physiology of insects. OUP. 10.1093/acprof:oso/9780199225941.001.0001

[bib35] Hotzy C., Arnqvist G. (2009). Sperm competition favors harmful males in seed beetles. Current Biology, 19(5), 404–407. 10.1016/j.cub.2009.01.045 19230665

[bib36] Iglesias-Carrasco M., Bilgin G., Jennions M. D., Head M. L. (2018). The fitness cost to females of exposure to males does not depend on water availability in seed beetles. Animal Behaviour, 142, 77–84. 10.1016/j.anbehav.2018.06.006

[bib37] Iglesias-Carrasco M., Taboada B., Lozano M., Carazo P., Garcia-Roa R., Rodriguez-Exposito E., Garcia-Gonzalez F. (2024). Sexual selection buffers the negative consequences of population fragmentation on adaptive plastic responses to increasing temperatures. Evolution, 78, 86–97. 10.1093/evolut/qpad193 37888875

[bib77] IPCC (2021). Climate change 2021: The physical science basis. Contribution of working group i to the sixth assessment report of the intergovernmental panel on climate change. Masson-delmotte V., Zhai P., Pirani A., et al. (Eds.). Cambridge University Press. 10.1017/9781009157896

[bib38] Jigisha, Iglesias-Carrasco M., Vincent A., Head M. L. (2020). Disentangling the costs of mating and harassment across different environments. Animal Behaviour, 165, 79–88. 10.1016/j.anbehav.2020.05.005

[bib39] Lale N. E. S., Vidal S. (2003). Effect of constant temperature and humidity on oviposition and development of Callosobruchus maculatus (F.) and *Callosobruchus subinnotatus* (Pic) on bambara groundnut, *Vigna subterranea* (L.) Verdcourt. Journal of Stored Products Research, 39(5), 459–470. 10.1016/S0022-474X(01)00028-5 11463395

[bib40] Le Galliard J.-F., Fitze P. S., Ferrière R., Clobert J. (2005). Sex ratio bias, male aggression, and population collapse in lizards. Proceedings of the National Academy of Sciences, 102, 18231–18236. 10.1073/pnas.0505172102 PMC1312374 16322105

[bib42] Llusia D., Márquez R., Beltrán J. F., Benítez M., Do Amaral J. P. (2013). Calling behaviour under climate change: Geographical and seasonal variation of calling temperatures in ectotherms. Global Change Biology, 19, 2655–2674. 10.1111/gcb.12267 23712567

[bib43] Londoño-Nieto C., Butler-Margalef M., García-Roa R., Carazo P. (2025). Temperature drives the evolutionary diversification of male harm in *Drosophila melanogaster* flies. Ecology Letters, 28(3), e70102. 10.1111/ele.70102 40111011

[bib44] Londoño-Nieto C., García-Roa R., González P., Carazo P. (2023). Thermal phenotypic plasticity of pre-and post-copulatory male harm buffers sexual conflict in wild *Drosophila melanogaster*. eLife, 12, e84759. 10.7554/eLife.84759 37102499 PMC10191624

[bib45] Mahgoup S. M., Zewar M. M., Dewidar O. (2019). Using thermal treatment to eliminate cowpea beetle *Callosobruchus maculatu*s (F.) infesting faba bean during storage and its effect on the physiochemical and technological properties. Egyptian Journal of Agricultural Research, 97(3), 645–662. 10.21608/ejar.2019.152569

[bib46] Małek D. K., Czarnoleski M. (2021). Thermal preferences of cowpea seed beetles (*Callosobruchus maculatus*): Effects of sex and nuptial gift transfers. Insects, 12(4), 310. 10.3390/insects12040310 33915679 PMC8066898

[bib47] Martinossi-Allibert I., Arnqvist G., Berger D. (2017). Sex-specific selection under environmental stress in seed beetles. Journal of Evolutionary Biology, 30, 161–173. 10.1111/jeb.12996 27749005

[bib48] McCaw B. A., Leonard A. M., Stevenson T. J., Lancaster L. T. (2024). A role of epigenetic mechanisms in regulating female reproductive responses to temperature in a pest beetle. Insect Molecular Biology, 33(5), 516–533. 10.1111/imb.12933 38864655

[bib49] McLeod D. V., Day T. (2017). Female plasticity tends to reduce sexual conflict. Nature Ecology & Evolution, 1(3), 0054. 10.1038/s41559-016-0054 28812713

[bib50] McNamara K. B., Sloan N. S., Kershaw S. E., Van Lieshout E., Simmons L. W. (2020). Males evolve to be more harmful under increased sexual conflict intensity in a seed beetle. Behavioral Ecology, 31(2), 591–597. 10.1093/beheco/arz186

[bib51] Nowakowski A. J., Watling J. I., Thompson M. E., Brusch G. A., Catenazzi A., Whitfield S. M., Kurz D. J., Suárez-Mayorga Á., Aponte-Gutiérrez A., Donnelly M. A. (2018). Thermal biology mediates responses of amphibians and reptiles to habitat modification. Ecology Letters, 21(3), 345–355. 10.1111/ele.12901 29314479

[bib52] Parker G. (1979). Sexual selection and sexual conflict. Sexual Selection and Reproductive Competition in Insects, 123, 166.

[bib53] Parker G., Partridge L. (1998). Sexual conflict and speciation. Philosophical Transactions of the Royal Society of London. Series B: Biological Sciences, 353, 261–274. 10.1098/rstb.1998.0208 9533125 PMC1692203

[bib54] Parrett J. M., Knell R. J. (2018). The effect of sexual selection on adaptation and extinction under increasing temperatures. Proceedings of the Royal Society of London B, 285, 20180303. 10.1098/rspb.2018.0303 PMC5936732 29669902

[bib55] Paukku S., Kotiaho J. S. (2005). Cost of reproduction in *Callosobruchus maculatus*: Effects of mating on male longevity and the effect of male mating status on female longevity. Journal of Insect Physiology, 51, 1220–1226. 10.1016/j.jinsphys.2005.06.012 16115646

[bib57] Perry J. C., Rowe L. (2018). Sexual conflict in its ecological setting. Philosophical Transactions of the Royal Society B: Biological Sciences, 373(1757), 20170418. 10.1098/rstb.2017.0418 PMC6125725 30150217

[bib56] Perry J. C., Garroway C. J., Rowe L. (2017). The role of ecology, neutral processes and antagonistic coevolution in an apparent sexual arms race. Ecology Letters, 20, 1107–1117. 10.1111/ele.12806 28683517

[bib58] Plesnar-Bielak A., Łukasiewicz A. (2021). Sexual conflict in a changing environment. Biological Reviews, 96(5), 1854–1867. 10.1111/brv.12728 33960630 PMC8518779

[bib59] Punzalan D., Rodd F. H., Rowe L. (2008). Sexual selection mediated by the thermoregulatory effects of male colour pattern in the ambush bug *Phymata americana*. Proceedings of the Royal Society of London B, 275, 483–492. 10.1098/rspb.2007.1585 PMC2596820 18089533

[bib60] R Core Team . (2014). R: A language and environment for statistical computing. R Foundation for Statistical Computing. ISBN 3-900051-07-0. http://www.R-project.org/(2014)

[bib61] Rankin D. J., Bargum K., Kokko H. (2007). The tragedy of the commons in evolutionary biology. Trends in Ecology & Evolution, 22, 643–651. 10.1016/j.tree.2007.07.009 17981363

[bib62] Rankin D. J., Dieckmann U., Kokko H. (2011). Sexual conflict and the tragedy of the commons. The American Naturalist, 177(6), 780–791. 10.1086/659947 21597254

[bib63] Reinhardt K., Naylor R. A., Siva-Jothy M. T. (2009). Ejaculate components delay reproductive senescence while elevating female reproductive rate in an insect. Proceedings of the National Academy of Sciences, 106(51), 21743–21747. 10.1073/pnas.0905347106 PMC2799855 19996174

[bib64] Rice W. R., Holland B. (1997). The enemies within: Intergenomic conflict, interlocus contest evolution (ICE), and the intraspecific Red Queen. Behavioral Ecology and Sociobiology, 41, 1–10. 10.1007/s002650050357

[bib65] Rodriguez-Exposito E., Garcia-Gonzalez F. (2021). Metapopulation structure modulates sexual antagonism. Evolution Letters, 5(4), 344–358. 10.1002/evl3.244 34367660 PMC8327942

[bib66] Rönn J., Katvala M., Arnqvist G. (2007). Coevolution between harmful male genitalia and female resistance in seed beetles. Proceedings of the National Academy of Sciences, 104(26), 10921–10925. 10.1073/pnas.0701170104 PMC1904142 17573531

[bib67] Savalli U. M., Fox C. W. (1999). The effect of male mating history on paternal investment, fecundity and female remating in the seed beetle *Callosobruchus maculatu*s. Functional Ecology, 13(2), 169–177. 10.1046/j.1365-2435.1999.00287.x

[bib68] Schielzeth H. (2010). Simple means to improve the interpretability of regression coefficients. Methods in Ecology and Evolution, 1(2), 103–113. 10.1111/j.2041-210X.2010.00012.x

[bib69] Tregenza T., Wedell N., Chapman T. (2006). Introduction. Sexual conflict: A new paradigm?. Philosophical Transactions of the Royal Society B: Biological Sciences, 361(1466), 229–234. 10.1098/rstb.2005.1796 PMC1569611 16612883

[bib70] Ursprung C., Den Hollander M., Gwynne D. T. (2009). Female seed beetles, *Callosobruchus maculatus*, remate for male-supplied water rather than ejaculate nutrition. Behavioral Ecology and Sociobiology, 63, 781–788. 10.1007/s00265-009-0711-z

[bib71] Van Lieshout E., McNamara K. B., Simmons L. W. (2014). Why do female *Callosobruchus maculatus* kick their mates?. PLoS One, 9(4), e95747. 10.1371/journal.pone.0095747 24752530 PMC3994112

[bib72] Vasudeva R. (2014). The influence of developmental temperature on sperm form and function in callosobruchus maculatus[Doctoral dissertation]. University of Lincoln.

[bib73] Wickham H., Chang W., Wickham M. H. (2016). Package ‘ggplot2’. Create Elegant Data Visualisations Using the Grammar of Graphics. Version, 2(1), 1–189.

[bib74] Yun L., Chen P. J., Kwok K. E., Angell C. S., Rundle H. D., Agrawal A. F. (2018). Competition for mates and the improvement of nonsexual fitness. Proceedings of the National Academy of Sciences, 115(26), 6762–6767. 10.1073/pnas.1805435115 PMC6042133 29891650

[bib75] Yun L., Chen P. J., Singh A., Agrawal A. F., Rundle H. D. (2017). The physical environment mediates male harm and its effect on selection in females. Proceedings of the Royal Society B: Biological Sciences, 284(1858), 20170424. 10.1098/rspb.2017.0424 PMC5524491 28679725

[bib76] Zajitschek S. R., Dowling D. K., Head M. L., Rodriguez-Exposito E., Garcia-Gonzalez F. (2018). Transgenerational effects of maternal sexual interactions in seed beetles. Heredity, 121(3), 282–291. 10.1038/s41437-018-0093-y 29802349 PMC6082829

[bib78] Zuk M., Garcia-Gonzalez F., Herberstein M. E., Simmons L. W. (2014). Model systems, taxonomic bias, and sexual selection: Beyond *Drosophila*. Annual Review of Entomology, 59(1), 321–338. 10.1146/annurev-ento-011613-162014 24160422

