# Supplementary material for: Temperature can reverse sexual conflict, facilitating population growth
Source: Evol Lett. 2025 Aug 1;9(5):558–66. doi: 10.1093/evlett/qraf022 (PMC12492134; doi:10.1093/evlett/qraf022)
Supplement: qraf022_Supplemental_File [file qraf022_supplemental_file.docx]

**Supplementary Information**

**Material and Methods**

Initial target sample sizes were of 42 focal females for all treatments except the control 'no harm', which included 50. However, due to unexpected contingencies final sample sizes used in the experimental analyses were as follows: at 24°C, low-intensity ‘harassment and mating’ = 38, high-intensity ‘harassment and mating’ = 39, low-intensity ‘harassment’ = 41, high-intensity ‘harassment’ = 39, ‘no harm’ = 47; at 28°C, low-intensity ‘harassment and mating’ = 40, high-intensity ‘harassment and mating’ = 40, low-intensity ‘harassment’ = 40, high-intensity ‘harassment’ = 39, ‘no harm’ = 49; and at 32°C, low-intensity ‘harassment and mating’ = 40, high-intensity ‘harassment and mating’ = 40, low-intensity ‘harassment’ = 42, high-intensity ‘harassment’ = 37, ‘no harm’ = 49.

**Results**

*Exploring interaction between temperature and sexual conflict for lifespan*

Although our study was primarily designed to examine female reproductive success across different experimental environments (see main manuscript), our data can also offer complementary information about whether temperature (24ºC, 28ºC or 32ºC) and sexual conflict treatments (i.e. ‘no harm’, low-intensity ‘harassment’, high-intensity ‘harassment’, low-intensity ‘harassment and mating’, and high-intensity ‘harassment and mating’) affect female lifespan. We found a significant interaction between temperature and sexual conflict treatments on female lifespan (F _8,605_=2.29, *p =* 0.02; Figure S2). Main effects of temperature (F _2,605_=274.5, *p <* 0.001) and sexual conflict treatment (F _4,605_=9.021, *p <* 0.001) were also significant. To explore this interaction, we analysed the data by temperature and found that sexual conflict treatment significantly affected female lifespan at all three temperatures. At 28ºC (F _4,203_=5.268, *p <* 0.001, estimate: -0.532 ± 0.115), we detected a strong decrease in lifespan of females in high-intensity ‘harassment and mating’ or high-intensity ‘harassment’ compared to those with ‘no harm’ (see Figure S2; Table S1). At 32ºC, sexual conflict treatment also affected female lifespan (F _4,203_=5.902, *p <* 0.001, estimate: -0.932 ± 0.084), but post hoc tests did not reveal significant effects between sexual conflict treatments (Figure S2; Table S1). At 24ºC, we detected a significant effect (F _4,199_=3.416, *p =* 0.009, estimate: 0.526 ± 0.135) driven by the lower lifespan of females exposed to high-intensity ‘harassment and mating’ compared to those under low-intensity ‘harassment and mating’ or low-intensity ‘harassment’ (Figure S2; Table S1).

We also examined the potential effects of temperature (24ºC, 28ºC or 32ºC) and sexual conflict on lifespan after pooling our data in low- and high-intensity sexual conflict treatments within the ‘harassment and mating’ and ‘harassment’ treatments (i.e. resulting in only three sexual conflict treatments: ‘harassment and mating’, ‘harassment’ and ‘no harm’) (Figure S2). This allowed us to increase statistical power and focus on differences driven by the type of sexual conflict experienced by females—specifically, whether they were exposed to both ‘harassment and mating’ or to ‘harassment’ alone. We obtained a significant interaction of temperature and sexual conflict treatment (F _4,611_=2.929, *p =* 0.02) on lifespan, as well as main effects of temperature (F _2,611_=268.11, *p <* 0.001) and sexual conflict treatment (F _2,617_=10.668, *p <* 0.001) (Figure S1). We then re-fitted statistical models separately for the three temperatures. At 28ºC, we found lifespan was affected by the sexual conflict treatment (F _2,205_=7.149, *p <* 0.001, estimate: -0.247 ± 0.083), with females under ‘harassment and mating’ and ‘harassment’ having lower lifespan than those under ‘no harm’ (Figure S2; Table S1). At 32ºC, the observed significant effect (F _2,205_=11.36, *p <* 0.001, estimate: -0.569 ± 0.059) was driven by females under ‘harassment’ or ‘no harm’ having a higher lifespan compared to those under ‘harassment and mating’ (Figure S2; Table S1). At 24ºC, we did not find an effect of the sexual conflict treatment on female lifespan (F _2,201_=2.039, *p =* 0.133).

*Exploring effects of number of males on male harm*

We conducted a complementary analysis to assess, in females continuously exposed to males throughout their lives, the potential effects of temperature and number of males (2 vs 1 males) on female reproductive success and lifespan. To this end, we fitted a linear model to explore the effects of temperature, number of males (i.e. high vs low sexual conflict) and their interaction, as fixed factors, on female lifetime reproductive success and lifespan. Because this analysis does not include the control treatment (‘no harm’, where females were kept alone), it cannot fully disentangle the effects of sexual conflict intensity per se, but it does offer complementary insight into whether the thermal environment and number of males influence female reproductive success and lifespan. Our results showed no interaction between temperature and the number of males on female lifetime reproductive success (F_2,454_ = 0.431, p = 0.650), but we found significant main effects for both temperature (F_2,454_ = 5.733, p = 0.003, estimate: –0.230 ± 0.080) and number of males (F_1,454_= 12.377, p < 0.001, estimate: –0.324 ± 0.092). Similarly, for female lifespan, the interaction between temperature and number of males was not significant (F_2,472_ = 2.205, p = 0.111), but we detected significant main effects of temperature (F_2,472_ = 237.108, p < 0.001, estimate: –0.684 ± 0.062) and number of males (F_1,472_ = 14.193, p < 0.001, estimate: –0.243 ± 0.064).

**Supplementary information**

**Table S1**. Results from post hoc tests assessing potential differences in the lifespan of *Callosobruchus maculatus* females across temperatures and sexual conflict treatments. Significant results are highlighted in bold, and marginally non-significant results are highlighted in italics. High: two males and one female (high intensity of sexual conflict; Low: one male and one female (low intensity of sexual conflict).

| Lifespan | 24ºC | | | | | 28ºC | | | | | 32ºC | | | | |
| --- | --- | --- | --- | --- | --- | --- | --- | --- | --- | --- | --- | --- | --- | --- | --- |
|  | Estimate | | | *t* | *P* | Estimate | | | *t* | *P* | Estimate | | | *t* | *P* |
| Harassment / Harassment and mating | **0,274** | **±** | **0,116** | **2,372** | **0,047** | 0,084 | ± | 0,115 | 0,732 | 0,745 | **0,363** | **±** | **0,115** | **3,156** | **0,005** |
| Harassment / No harm | 0,184 | ± | 0,133 | 1,381 | 0,351 | **-0,407** | **±** | **0,132** | **-3,091** | **0,006** | 0,001 | ± | 0,132 | 0,011 | 0,999 |
| Harassment and mating / No harm | -0,090 | ± | 0,134 | -0,675 | 0,778 | **-0,491** | **±** | **0,131** | **-3,738** | **0,001** | **-0,361** | **±** | **0,131** | **-2,749** | **0,017** |
| Harassment and matingHigh / Harassment and matingLow | **-0,520** | **±** | **0,163** | **-3,185** | **0,013** | -0,400 | ± | 0,160 | -2,501 | 0,092 | 0,066 | ± | 0,160 | 0,496 | 0,999 |
| Harassment and matingHigh / HarassmentLow | -0,386 | ± | 0,162 | -2,384 | 0,121 | -0,226 | ± | 0,161 | -1,400 | 0,628 | -0,299 | ± | 0,163 | -1,830 | 0,357 |
| Harassment and matingHigh/ HarassmentLow | **-0,668** | **±** | **0,160** | **-4,172** | **0,000** | -0,341 | ± | 0,160 | -2,133 | 0,207 | *-0,419* | *±* | *0,158* | *-2,648* | *0,063* |
| Harassment and matingHigh / No harm | -0,347 | ± | 0,155 | -2,237 | 0,168 | **-0,691** | **±** | **0,153** | **-4,532** | **0,000** | -0,361 | ± | 0,153 | -2,368 | 0,126 |
| Harassment and matingLow / HarassmentLow | 0,133 | ± | 0,163 | 0,816 | 0,926 | 0,175 | ± | 0,161 | 1,085 | 0,814 | -0,299 | ± | 0,163 | -1,830 | 0,357 |
| Harassment and matingLow / HarassmentLow | -0,148 | ± | 0,161 | -0,921 | 0,889 | 0,059 | ± | 0,160 | 0,368 | 0,996 | *-0,419* | *±* | *0,158* | *-2,648* | *0,063* |
| Harassment and matingLow / No harm | 0,173 | ± | 0,156 | 1,106 | 0,803 | -0,291 | ± | 0,153 | -1,908 | 0,314 | -0,361 | ± | 0,153 | -2,368 | 0,126 |
| HarassmentHigh/ HarassmentLow | -0,282 | ± | 0,160 | -1,759 | 0,399 | -0,116 | ± | 0,161 | -0,720 | 0,952 | -0,120 | ± | 0,161 | -0,744 | 0,946 |
| HarassmentHigh / No harm | 0,040 | ± | 0,155 | 0,255 | 0,999 | **-0,466** | **±** | **0,154** | **-3,032** | **0,021** | -0,062 | ± | 0,156 | -0,400 | 0,995 |
| HarassmentLow/ No harm | 0,321 | ± | 0,153 | 2,099 | 0,222 | -0,350 | ± | 0,153 | -2,294 | 0,148 | 0,058 | ± | 0,150 | 0,383 | 0,995 |


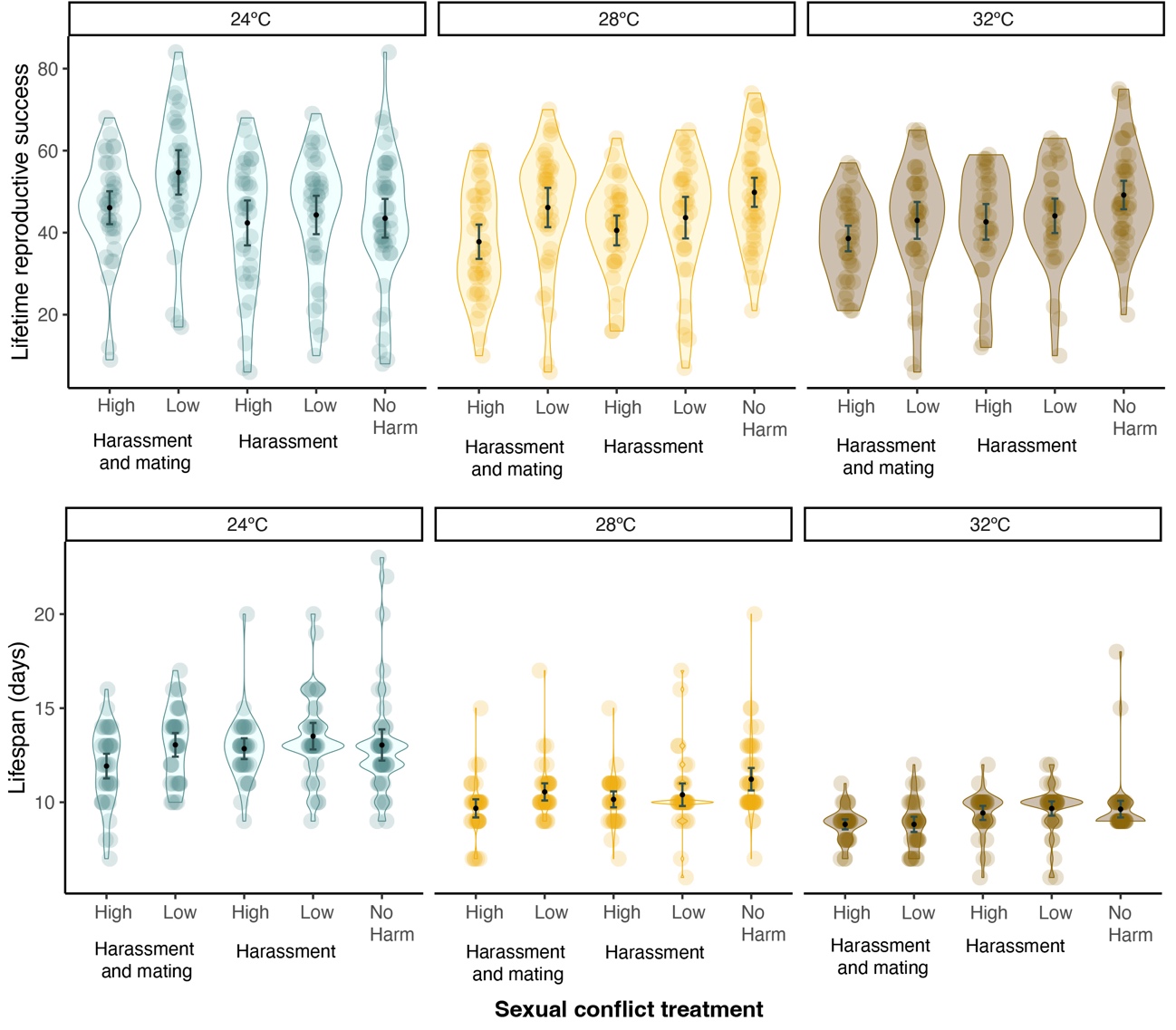


**Figure S1**. Plots for distribution data in lifetime reproductive success (top) and lifespan (bottom) of *Callosobruchus maculatus* females (black dots and bars indicate mean ± SEM) exposed at three different temperatures (24°C, 28°C or 32°C). Both variables are shown according to the sexual conflict treatment (‘Harassment and mating’, ‘harassment’ and ‘no harm’) and its intensity (high-intensity: two males and one female; low-intensity: one male and one female; and no harm: females alone). All focal females had a preliminary stage in which they were allowed to mate with one male for 1.5 hours at 28°C.


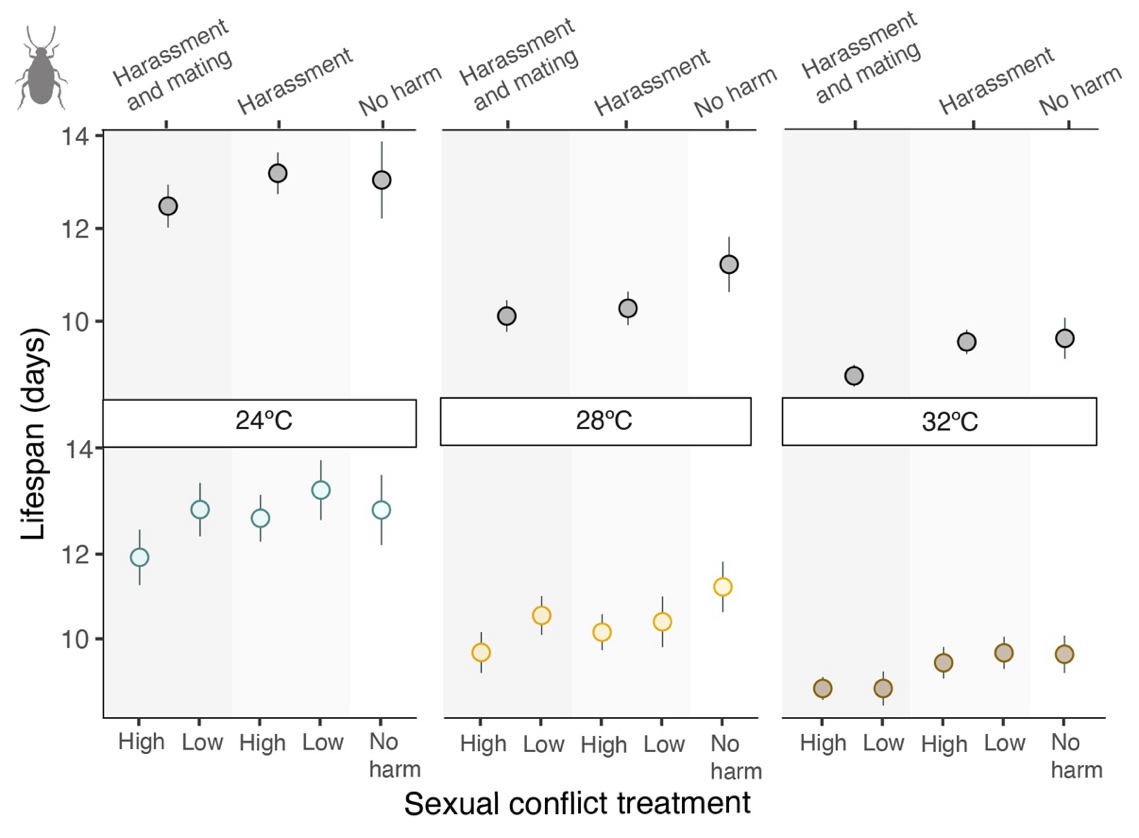


**Figure S2.** Plots for lifespan of *Callosobruchus maculatus* females (mean ± SEM) exposed at three different temperatures (24°C, 28°C or 32°C). Lifespan according to the sexual conflict treatment (‘Harassment and mating’, ‘harassment’ and ‘no harm’; top plot) and its intensity (high-intensity: two males and one female; low-intensity: one male and one female; and no harm: females alone; bottom plot) is shown. The top plot shows the results from the combination of intensity treatments. All focal females had a preliminary stage in which they were allowed to mate with one male.
